# Short Barcodes for Next Generation Sequencing

**DOI:** 10.1371/journal.pone.0082933

**Published:** 2013-12-30

**Authors:** Katharina Mir, Klaus Neuhaus, Martin Bossert, Steffen Schober

**Affiliations:** 1 Institute of Communications Engineering, Ulm University, Ulm, Germany; 2 Chair for Microbial Ecology, Technische Universität München, Freising, Germany; Tel Aviv University, Israel

## Abstract

We consider the design and evaluation of short barcodes, with a length between six and eight nucleotides, used for parallel sequencing on platforms where substitution errors dominate. Such codes should have not only good error correction properties but also the code words should fulfil certain biological constraints (*experimental parameters*). We compare published barcodes with codes obtained by two new constructions methods, one based on the currently best known linear codes and a simple randomized construction method. The evaluation done is with respect to the error correction capabilities, barcode size and their experimental parameters and fundamental bounds on the code size and their distance properties. We provide a list of codes for lengths between six and eight nucleotides, where for length eight, two substitution errors can be corrected. In fact, no code with larger minimum distance can exist.

## Introduction

Modern high-throughput techniques for DNA sequencing also allow to sequence RNA of different independent samples during a single run. For this purpose, the cDNA molecules of each sample are *tagged* with a unique sequence, the *code word*, and then pooled into one single library [1]. We refer to a set of such code words as a *barcode*. Using the code words, the *reads* obtained by the sequencing procedure can be *demultiplexed* afterwards, i.e., they are assigned to the different samples.

Due to errors occurring during the library preparation and the sequencing process a *cross-talk* event may occur, where reads are assigned to the wrong sample. This is especially of importance when a gene is very differently transcribed between two samples. To avoid cross-talk, a careful design of the barcode is required. Clearly, the design depends on the sequencing platform. For example, on the Roche 454 [2] the predominant type of errors are insertions and deletions (indels) [3], while on the Illumina sequencing platforms [4] the most frequent errors are substitutions (see for example [5]). Further constraints that need to be considered for experimental reasons are, e.g., GC-content and homopolymer lengths of the code words. Biased GC or long homopolymers increase the error rates in the enzymatic processes used.

Many Barcode designs are based on algebraic codes like binary Hamming codes [1], codes over quaternary alphabets (over the ring 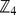) [6] or BCH-codes [7]. Such algebraic constructions are not only providing a way to construct the codes, but they usually also provide efficient techniques for *decoding* (or *demultiplexing*). But it is interesting to note that many currently used barcodes have a rather short length 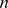, defined as the number of nucleotides used to compose each single code word. For example, the barcode used in some Illumina's TrueSeq Kits has length 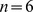 (with a size of 48 code words). But for such short and small codes decoding can be implemented in a simple table, providing a decoding algorithm that is optimal (maximum likelihood decoding, see below), without needing the algebraic structure of the code. Further it becomes possible to employ search algorithms to construct codes for example the *barcrawl* algorithm [8] which has a time complexity exponential in 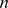 (but it is worth noting that searching through all possible codes appears to be impossible as the number of possible codes grows double exponentially with 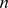).

In this work, we search for the best possible barcode for a given set of experimental constraints. We propose two new constructions, the first uses the database of the currently best known linear codes [9] and the second is a simple random search strategy. We compared them with currently known barcodes regarding their error correction properties (such as minimum distance, distance distribution, and error probabilities) and their experimental parameters such as GC-content and homopolymer length. The results are presented in a list of codes that can be readily used for applications.

The outline of the paper is as follows: The *Methods* section discusses multiplexing and demultiplexing and deals with barcode design; in the *Results* section barcodes of different designs are compared, and conclusions are given in the section *Discussion and Conclusions*.

## Methods

### Parallel Sequencing

**Figure 1:**
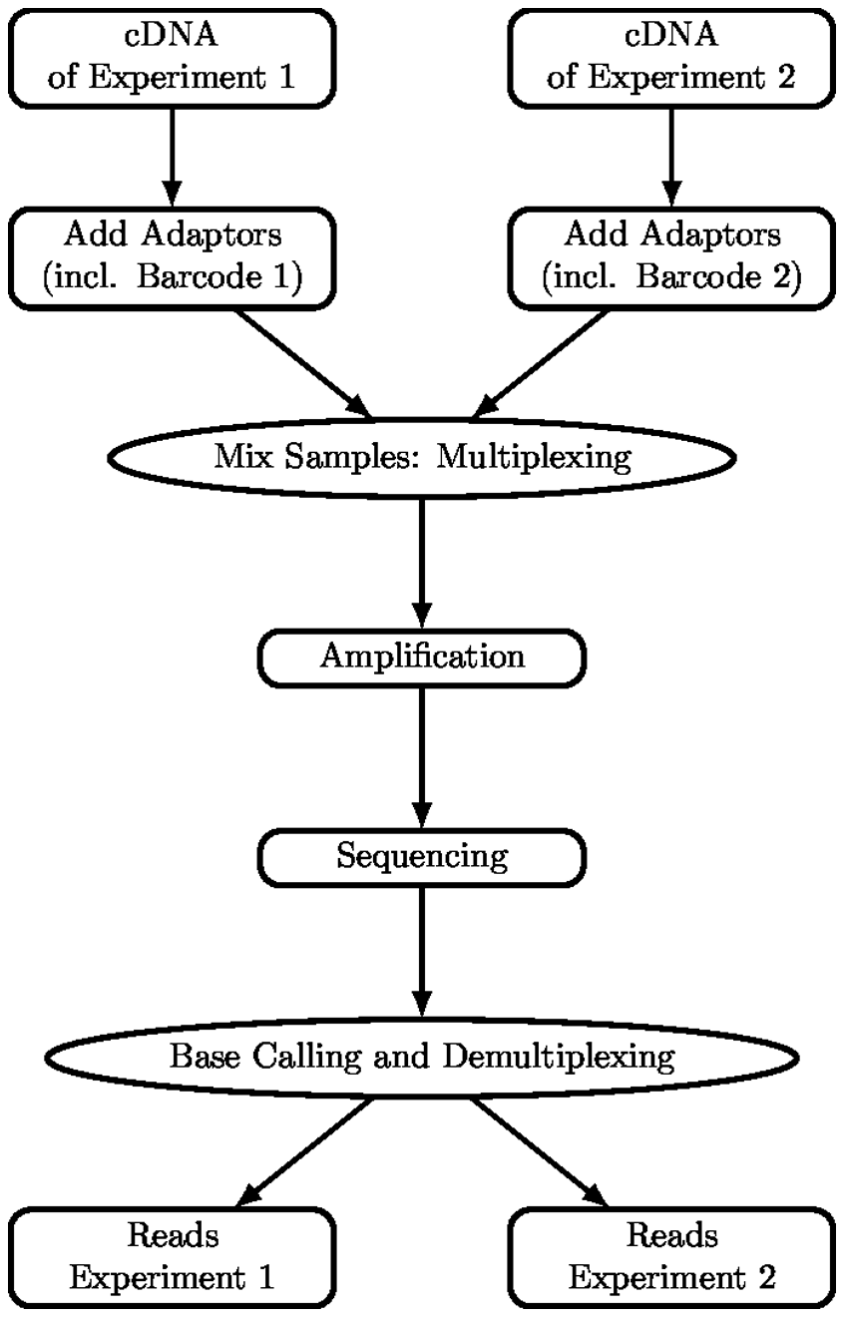
Schematic view. Illumina protocol for parallel sequencing of RNA samples as example.

A schematic view on the protocol is shown in Figure 1 according to protocols from Illumina. For each sample, the adapters are ligated to the cDNA. These adapters include a unique code word of length 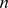 nucleotides, chosen out of a predefined (barcode) set 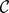, which identifies the corresponding sample. Demultiplexing is performed after bridge amplification and sequencing. Each read obtained by the sequencer has to be assigned to one of the samples.

Errors are induced at any step of this process, for example during

1. the PCR (amplification),
2. the bridge amplification, and
3. sequencing, since misreads occur.

For example, PCR can be employed using the *Taq* polymerase, which induces reported single base substitutions at a rate of 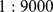, and one indel out of 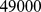 bases [10]. Other polymerases with less errors exist, however, Illumina uses a proprietary enzyme mix of unknown error rate.

The predominant errors after *base calling* are substitutions occurring at a rate of roughly 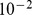 to 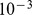, whereas indels are reported at rates of roughly 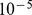 to 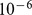, see [5], [11] (this further depends on the Q-value filters used [5]). It is known that the error rate varies with the position in the read. Whether there is a higher error rate in the context of certain motifs (e.g., GCC) remains undecided, [5], [12].

### System Model and Coding

The design of schemes for multiplexing and demultiplexing depends on the sequencing technique. As mentioned, we focus on the most widely used technique employed by Illumina.

### A Communciation Theoretic Model of Barcoding

**Figure 2:**
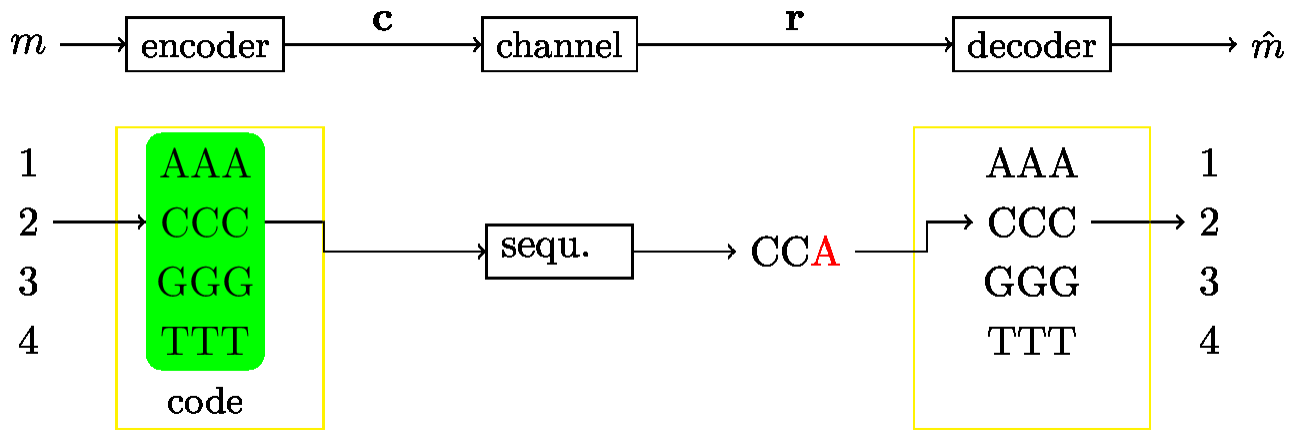
Communication theoretic model. In the upper part of the figure the general model is shown, while in the lower part an example is depicted: In particular the messages consist of the numbers 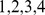 which are encoded using the code shown in the green box. In the example the number 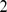 is encoded with the code word 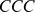. On the receiver side, 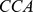 is received, which is decoded to the number 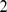. Notice, that this decoding procedure is only rational if we assume that one error is more likely than two errors.

In order to demultiplex, the code word is extracted from the read and used to assign the read to one of the samples. In communication theoretic notation, this can be interpreted as the problem to transmit a message over a noisy channel (upper panel of Figure 2). In particular, an integer number 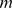, chosen from the set of possible messages 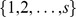, where 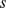 is the number of samples, should be communicated to the *receiver* as follows: The number 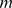 is *encoded* with a word of length 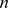 chosen from a *code* 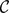 and send over the *noisy channel*. For our purpose a block code is defined as follows.

#### Definition 1

*A (block) code* 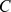 *is a subset of* 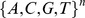 *, where* 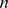 *is the code length,* 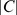 *is the code cardinality and* 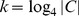 *is the code dimension. The ratio* 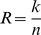 *is called the rate of the code.*

The channel is a mathematical model of the sequencing process. If we assume that only substitution errors occur (a valid assumption, since indels occur about 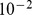 to 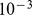 less likely), we can describe the process using the conditional probability to receive 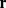, given that a code word 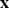 was sent, i.e., by 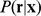 for 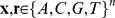. On the receiver side the received word 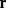 is used to give an estimate of the message which we denote 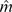. If we assume that no error occurred, 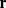 is an element of the code 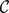 and identifies the message 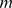 unequivocally. But if 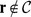, we need to employ a rule for read assignment. In accordance with coding theoretic notions, we will refer to such a rule as *decoder*, which will be formally defined later.

Suppose, we have a given channel, the ultimate goal of the design of the communication system is to provide a code and a decoder that minimizes the error probability. To this end, we consider different types of errors:

#### Definition 2

*Assume that the probability to choose a message* 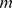 *out of* 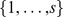 *is* 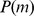 *and that* 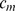 *is the corresponding code word. The* ***average decoding*** *error probability is defined as*

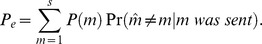
The ***maximum error*** is defined as
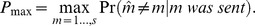



In general, the error probabilities defined above depend on (i) the channel, (ii) the code and (iii) the prior probabilities of the messages. Shannon [13], in his seminal work, showed that the average decoding error can be made arbitrarily small provided that a sufficiently long code is chosen and that its rate is smaller than the so called *capacity* of the channel. The latter is a quantity depending on the conditional probabilities describing the channel only. Note that these (and other) results do not provide a lower bound on the average error that can be reached with finite code lengths (but see for example Gallagher [14] for results that provide a connection between the rate of convergence of 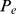 and the code length 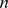).

However, it is important to note that the performance of systems crucially depends on the channel, which guides the design of the code and the decoder. In the following we will introduce the channel model we use first, and discuss the possible decoding principles next.

#### Channel Model

For the system design, a simplified channel is employed to model the sequencing process of the Illumina platform. First, we will design the system assuming that only substitution errors occur, since indels occur about 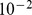 to 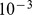 less likely. Further, it is assumed that errors occur independently of each other and independent of the position with rate 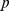 (although it is known that the error rate is cycle dependent in general, but on a short range of the barcodes such assumption appears to be reasonable [5]). Finally, we assume that all possible substitutions are equally likely, although different rates have been reported by Kao et al. [15]. Nevertheless, as it will be shown later, a code designed under the assumption of equally likely substitutions still provides a good performance if used over the channel reported in [15] (see also the *Results* part).

In coding theory such an error model is known as a 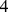-ary symmetric and memoryless *channel* [9]. We formally describe the channel by the probability to obtain a received word 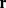 given that a code word 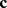 was chosen, i.e., with 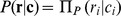.

For evaluation purposes, we again assume a memoryless channel, but this time we allow different transition probabilities between different nucleotides, following the statistical analysis of [15]. The transition probabilities are shown in Table 1.

**Table 1:** Transition probabilities (76-cycle Ga-II, phiX173, Bustard [15]).

	A	C	G	T
A	0.98896	0.00337	0.00296	0.00470
C	0.00877	0.97716	0.00336	0.01071
G	0.00485	0.00252	0.98617	0.00646
T	0.00289	0.00517	0.00665	0.98529

#### Decoding Principles

For a certain channel model, we can employ several decoding principles. In general, a decoder should select a word 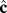 which maximizes the posterior probability, i.e.,
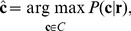
(1)see [14]. Note that
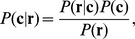
and that 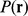 is independent of the decoding rule. Further, if all codewords are equally likely, i.e., 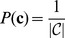 (this assumes that the different samples have equal size *after* the PCR, since the cDNA libraries with the different barcodes are mixed in equimolar amounts), maximizing the right hand side of Eq. (1) is equivalent to the *maximum likelihood decoding rule* (ML decoding)
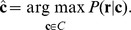



For the discrete memoryless q-ary symmetric channel considered here, the maximum likelihood decoding rule simplifies to the so called *minimum distance decoding*. To this end, recall that the Hamming distance 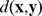 of two sequences 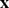 and 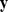 with equal length is defined as the number of differing positions. The minimum distance decoding rule is given by
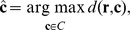
i.e., maximum likelihood decoding is obtained by choosing the codeword that is closest to the received word (see [17]).

It may happen that there are several codewords with the same distance to the received word. In this case, there are different possibilities to proceed. First, one of the possible codewords is chosen at random. Second, so called *list decoding* procedures will give a list of possible codewords. Here, we follow a third possibility and declare a *decoding failure* if there is no unambiguous decision possible. In NGS, millions of reads are obtained, and a decoding failure causes the drop-out of only very few reads from the total read amount. However, in the case of genes weak in one condition and strong in another, decoding with random codeword replacement would cause a possible substantial cross-talk. For later reference, the decoder is denoted as the function
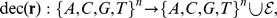
where 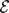 denotes a decoding failure.

In general, implementing a ML or minimum distance decoding rule is practically impossible. For a large number of code words it is computationally prohibitive to check all possible code words. However, in our case the number of code words is small, therefore, it is possible to implement a minimum distance decoder, using a table of all possible received words together with the closest code word. We require a table of size roughly 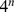, further, lookups can be implemented using hash tables.

### Constraints on Barcodes

The design of a barcode is governed by experimental, coding theoretic, but also financial constraints. The cardinality and length of the barcode is limited by the simple fact that all words in the set have to be synthesised, which is a money consuming process. Further, longer barcodes reduce the amount of useful read-lengths. The barcode has to be designed with respect to the error model to allow the correction of possible errors (this is discussed below in more detail). In addition, we need to consider the experimentally motivated constraints, like for example the GC-content or the homopolymer length, see the discussion below.

#### Coding Theoretic Constraints

In general, the *distance* between the code words should be as large as possible, but the optimal distance measure is not obvious. As discussed in the previous section, minimum distance decoding is the optimal choice assuming a symmetric channel and messages equally likely. Therefore, we will concentrate on codes with large minimum Hamming distance, which we define as follows:

#### Definition 3

*The Hamming distance* 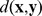 *between two words* 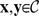 *is the number of positions* 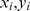 *that are different in two code words. The minimum distance of a code* 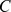 *is the smallest Hamming distance between any pair of two codewords, or more formally*

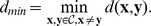



The minimum distance is related to the number of substitution errors which can be guaranteed to be corrected. Namely, using a code with minimum distance 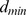 at least 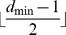 substitution errors can be corrected for each code word.

It is quite obvious that for a given length 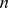 and a code cardinality 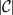 (or equivalently, given the code dimension 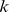), the Hamming distance can not be arbitrarily large. In fact, there are several bounds on the cardinality of a code given its minimum distance. The maybe simplest one is the following:

#### Fact 1 (Singleton bound)

*Let* 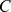 *be a code with length* 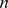 *and minimum distance* 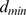 *. Then*

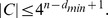



The following limit gives a bound on the code cardinality. It depends on the numbers of error that can be guaranteed to be corrected:

#### Fact 2 (Hamming bound (for alphabet size 4))

*Let* 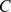 *be a code with length* 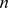 *and* 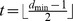 *, which is the number of substitution errors that can be guaranteed to be corrected. Then*

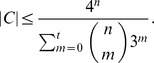



Different codes having the same minimum distance can have different error correction capabilities, as they may have different *distance distributions*, e.g. [16]. To this end we give the following definition.

#### Definition 4

*For a code* 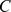 *with length* 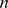 *, the distance distribution* 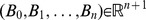 *is defined by*

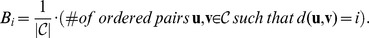



**Figure 3:**
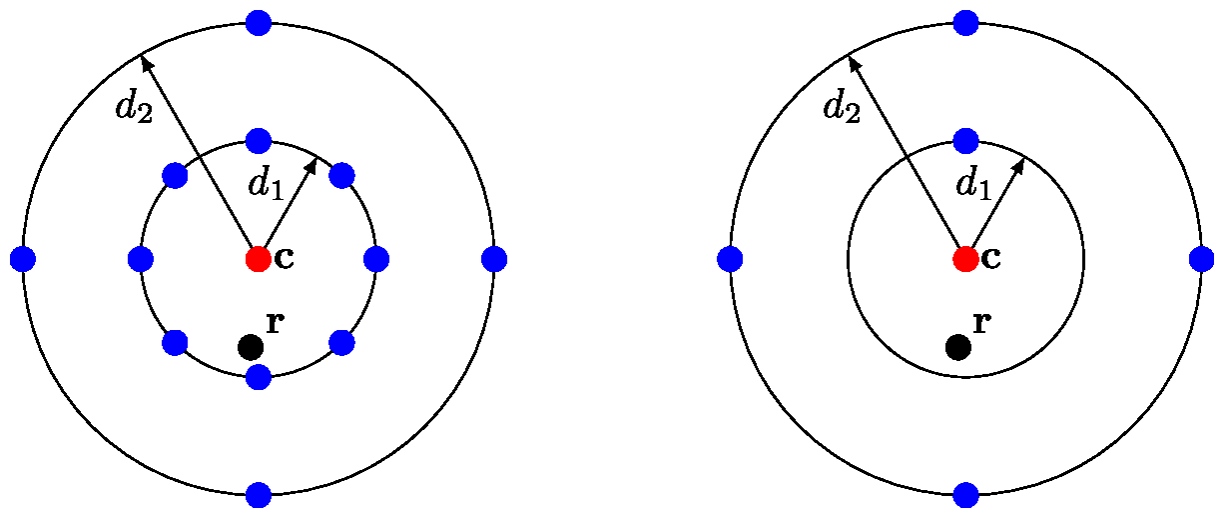
Influence of distance distribution. Illustration of codes having the same minimum distance but different distance distributions. The red dot represents the word sent, the black dot the received word 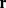, the blue dots are other codewords. In both cases, there is at least one code word with distance 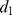 (hence the minimum distance). On the right hand side 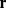 can be correctly decoded and assigned to 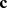, while on the left hand side 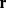 is assigned to the wrong codeword (the closest blue dot below 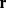).

Notice that 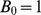 and 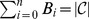, and that the number 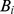, quantifies the number of code words at distance 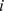 for an *average* code word. The effect of the distance distribution on the error correction capability is illustrated in Figure 3.

#### Experimental Constraints

Beside the coding theoretic constraints discussed, a barcode has to be designed with respect to experimental constraints, like GC-content and homopolymer length. The GC-content of a code word quantifies the number of 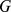 and 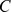 in the sequence. GC-rich parts like to form secondary structures if they are present in a single strand and do not open (melt) in a double strand easily. Both phenomena cause enzyme stalling or drop-off at GC-rich sequences. The homopolymer length, denoted with 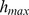, is the longest uninterrupted repetition of the same base in a given sequence. Homopolymers cause enzyme slipping, thus indels. Indeed, in [5] an increase of the insertion probability with a growing homopolymer length is reported.

### Construction of Codes

In the following we will discuss two different methods to construct barcodes. The first is based on the currently best linear code, while the second uses a simple random construction. The second method allows to directly include the experimental parameters into the construction process, while the first method does not allow this. Hence, in the latter case the constructed code has to be adapted to the experimental parameters by expurgating code words that do not match the constraints.

#### Linear Codes

Linear codes appear to be quite popular for barcodes, e.g., [1], [7]. In order to give a formal definition, we need to map the nucleotide letters to the elements of a finite field 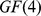 to define an addition and multiplication operation. For convenience, we will skip the technical details here and define addition and multiplication on 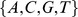 according to Table 2. For vectors in 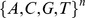 addition is defined by a point wise application of the addition defined above, namely
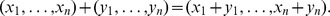
Now we can define a linear code as follows:

**Table 2:** Addition and multiplication defined on {*A*,*C*,*G*,*T*}.

+	A	C	G	T
A	A	C	G	T
C	C	A	T	G
G	G	T	A	C
T	T	G	C	A

⋅	A	C	G	T
A	A	A	A	A
C	A	C	G	T
G	A	G	T	C
T	A	T	C	G

#### Definition 5 (Linear code)

*A linear code* 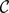 *of length* 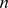 *and dimension* 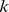 *is block code (Definition 1) over* 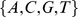 *such that for any codewords* 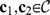


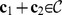
and for any 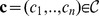 and any 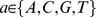


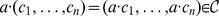
where addition and multiplication on 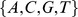 are defined according to Table 2.

The algebraic structure of the code can be exploited to design efficient decoding algorithms, e.g. [17]. In fact, much work in the last decades has focused on the construction and design of decoding algorithms for linear codes.

In important property of linear codes is that the distance distribution coincides with its *weight distribution*, which is defined as follows (e.g. [16]).

#### Definition 6

*For a code* 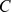 *with length* 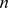 *, the weight distribution* 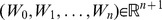 *counts the number of code words* 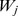 *with weight* 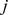 *, where the weight of a code word is the number of nonzero elements.*

In order to construct linear codes over 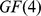 with good minimum distance, we use the database of the currently best known linear codes [9], [18]. To access the database and to construct the corresponding code, we use the computer algebra system MAGMA [19]. An overview of the algorithms used to determine code tables is given in [18].

#### Randomized Construction of Codes

To construct a barcode of length 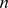 the barcrawl algorithm [8] starts from the complete list of all 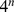 possible code words and then it successively removes code words that do not match the experimental constraints and minimum distance properties. In contrast, our algorithm starts with an empty code and successively adds new code words in a greedy way (regarding the distance): Suppose a code with prescribed length, cardinality, maximal homopolymer length 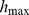, and upper and lower bounds on the GC-content should be constructed. Assume for now that the code should have minimum distance 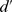. We first create a list 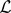 of all possible words of length 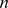, that match the homopolymer length and the GC-content limits. For this list, we compute the Hamming distances 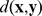 for all pairs 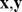 (this has to be conducted only once). Both, 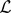 and 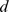, are passed to Table 3. We obtain a potential barcode 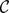, and, if its cardinality is large enough, the process is stopped. Otherwise, the algorithm is repeated, or, if no code can be found, 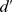 is lowered by 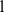 to search for a code with a smaller minimum distance.

**Table 3:** Algorithm RndBarcode.

1: RndBarcode 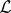 , 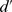 , 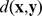
2: 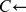 list containing a random chosen 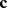 from 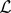
3: remove 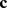 from 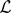
4: **while** 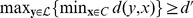 **do**
5: 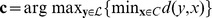
6: remove 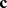 from 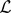 and add 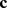 to 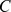
7: **end while**.
8: **end procedure**.

Let us note, that the algorithm has an exponential complexity in 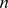. We need to store the Hamming distances between all words matching the experimental constraints (which requires to store 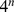 entries, due the symmetry of the Hamming distance) and in line 4 of Table 3 we have to make exponentially many comparisons (in the order of 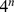). Hence, similar to the barcrawl algorithm, we may use this approach only for small 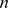.

## Results

In the following, we compare our randomly drawn barcodes, RN(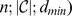 (each code is the best found in 1000 trials) with published barcodes. First, we compare with barcodes included in Illumina's TrueSeq Kits (Oligonucleotide sequences  2007–2011 Illumina, Inc. All rights reserved.) with size 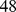 and length 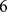, which we denote with IL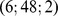. We further consider the codes proposed by Bystrykh (see Information S1 Table S2 (Pages 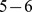) in [6]) of different lengths which are denoted with BY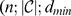) and the codes obtained by barcrawl with BC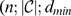). Finally, we consider the best known linear codes, BL(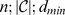) over 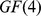 of different lengths 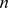 and sizes 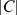. Note that the dimension is 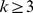 in order to get enough codewords.

### Basic code properties

For each code length 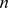 and minimum distance 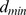, we fixed the experimental constraints on the homopolymer length 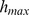 and the GC-content range of the code words. We are now interested in the largest possible set size that can be determined if all code words are deleted that do not fit the constraints. The basic code properties are summarized in Table 4. The last column assesses the different code sizes. For a fairly realistic evaluation of the different barcodes, we evaluated the codes using a channel with the transition probabilities shown in Table 1 [15], where we consider the average (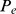) and the maximum error (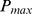) specified in Definition 2.

**Table 4:** Properties of barcode sets with fixed experimental constraints. The average (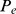) and the maximum error (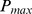) are obtained over a non-symmetric channel defined in the *Channel Model* part.

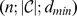	*n*	*d_min_*	GC [%]	*h_max_*	*P_e_*	*P_max_*	Comment
IL(6;48;2)	6	2	0–83.3	4	0.003720	0.024100	
BY(6;13;3)	6	3	50	1	0.00115744974058	0.00152675606062	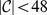
RN(6;48;3)	6	3	50	1	0.00293720069404	0.00372112063192	Largest set size
BC(6;45;3)	6	3	50	1	0.00293934865265	0.00433319686588	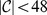
BY(6;48;3)	6	3	33.3–66.7	2	0.00287479186204	0.00344409742315	
RN(6;91;3)	6	3	33.3–66.7	2	0.00336953649051	0.00498588334159	Largest set size
BC(6;90;3)	6	3	33.3–66.7	2	0.00333779055118	0.00479221839011	
BL(6;0;4)	6	4	50	1	not calc.	not calc.	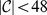
RN(6;20;4)	6	4	50	1	0.00160381775190	0.00193212583882	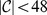
BC(6;21;4)	6	4	50	1	0.00170852113961	0.00223078114371	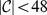
BL(6;60;4)	6	4	33.3–66.7	2	0.00350044276484	0.00415971952076	Largest set size
RN(6;22;4)	6	4	33.3–66.7	2	0.00154681909096	0.00257664771207	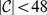
BC(6;27;4)	6	4	33.3–66.7	2	0.00213145291469	0.00308957132629	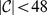
BY(7;52;3)	7	3	42.9–57.1	1	0.00243615939854	0.00317400700813	
BL(7;34;3)	7	3	42.9–57.1	1	0.00145478099500	0.00234874403316	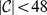
RN(7;131;3)	7	3	42.9–57.1	1	0.00398044428796	0.00512316866215	
BC(7;134;3)	7	3	42.9–57.1	1	0.00409145276126	0.00585134792272	Largest set size
BL(7;29;4)	7	4	42.9–57.1	2	0.00088948621485	0.00192453915606	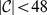
RN(7;61;4)	7	4	42.9–57.1	2	0.00218757478563	0.00410906150349	
BC(7;67;4)	7	4	42.9–57.1	2	0.00246956732624	0.00427006271525	Largest set size
BL(7;41;4)	7	4	28.6–71.4	2	0.00122876771354	0.00234288468511	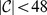
RN(7;63;4)	7	4	28.6–71.4	2	0.00215726162765	0.00325896653775	
BC(7;71;4)	7	4	28.6–71.4	2	0.00270966900931	0.00398044085558	Largest set size
BL(7;62;4)	7	4	14.3–85.7	3	0.00188935248825	0.00299764003488	
RN(7;68;4)	7	4	14.3–85.7	3	0.00229868424521	0.00375703185561	
BC(7;78;4)	7	4	14.3–85.7	3	0.00291964205936	0.00482760240470	Largest set size
BY(8;52;4)	8	4	50	1	0.00138438837587	0.00174725711803	
BL(8;8;4)	8	4	50	1	0.00017211463306	0.000435829557311	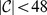
RN(8;90;4)	8	4	50	1	0.00174036590003	0.00250377449382	
BC(8;97;4)	8	4	50	1	0.00208718229848	0.00348552721763	Largest set size
BL(8;50;5)	8	5	37.5–62.5	3	0.000164930826698	0.000271101846767	Largest set size
RN(8;46;5)	8	5	37.5–62.5	3	0.000151706895645	0.000246945726731	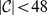
BC(8;50;5)	8	5	37.5–62.5	3	0.000164977426615	0.000263832223954	Largest set size
BL(8;58;5)	8	5	25–75	3	0.000185250799755	0.000305507449322	Largest set size
RN(8;48;5)	8	5	25–75	3	0.000161049267064	0.000239630273434	
BC(8;56;5)	8	5	25–75	3	0.000175939065403	0.000277667990980	

### Codes with length 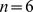


The barcode properties for length 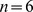 are summarized in Table 4. The Illumina barcode has the lowest minimum distance, the largest homopolymer length and the widest range of GC-content. All other codes show an improvement in their properties. Comparing the best linear code with our RN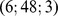, we see that BL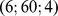 has a larger set and a better minimum distance but worse experimental parameters. If we relax the experimental constraints, the set size is enlarged for RN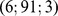.

The largest minimum distance for 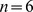 is 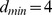. In fact, this minimum distance can not be improved as shown by the Singleton bound. If we assume a minimum distance of 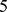, the code size is upper bounded by 16 (see Fact 1).

**Figure 4:**
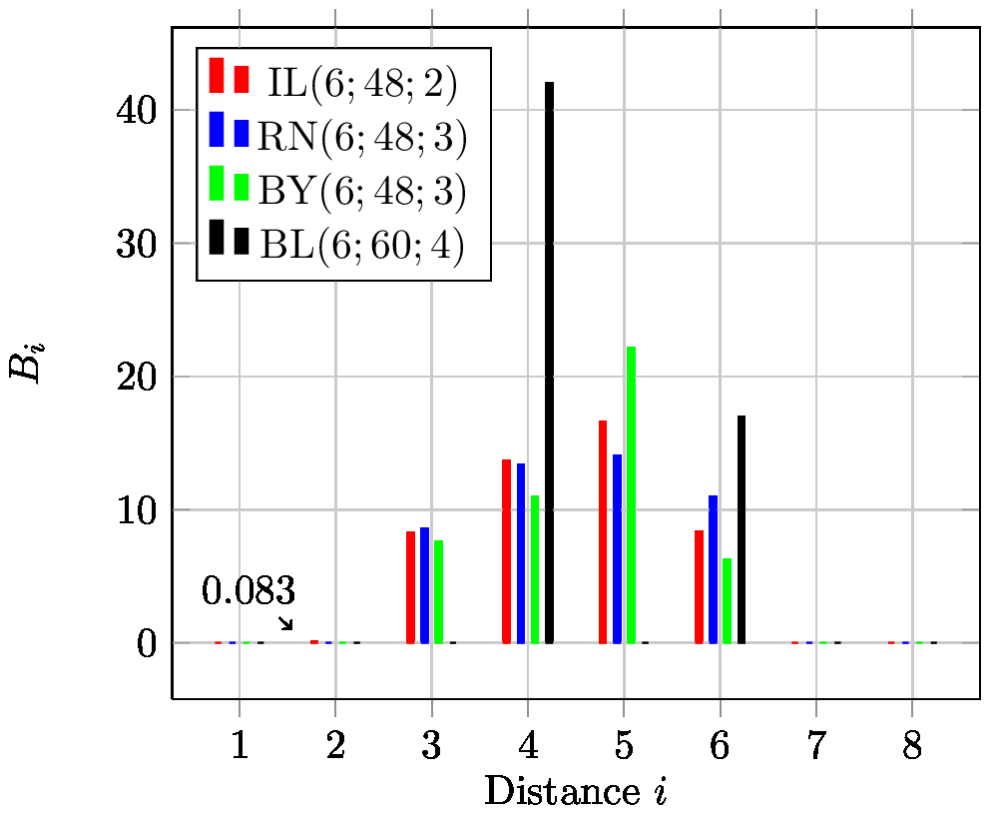
Distance distribution. Comparison of selected barcodes of length 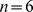.

If we compare average error during the transmission over a non-symmetric channel (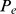 in Table 4) codes with a cardinality of 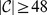 show a comparable performance. Interestingly, the increased minimum distance of four does not provide a significant improvement in comparison with codes having minimum distance three. Also, the Illumina barcode, having only a minimum distance of two, shows only a slightly worse error probability compared to all other codes. But, if we take a closer look at the *maximum error* 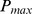 of the Illumina set IL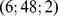 it is a magnitude worse in comparison with all other codes of length 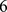. This is due to the fact that there are pairs of codewords that are quite close, which becomes clear if we compare the distance profiles (specified in Definition 4) in Figure 4. Actually, IL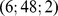 has a comparable distance distribution as BY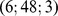 and RN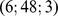. Closer inspection of the distribution reveals that 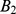 (see Definition 4) is only 0.083.

### Codes with length 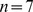


For barcodes of length 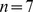, the best experimental parameters together with the largest set size, are achieved for BC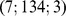. It is guaranteed that the codes with length 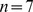 and 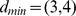 can correct one substitution error, the same as for 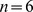. Regarding the minimum distance, there maybe room for an improvement. Namely, the Singleton bound allows a minimum distance of five, and, inspecting the Hamming bound, also shows that the guaranteed correction of two errors might be possible, which implies that 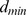 is equal to five.

### Codes with length 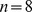


If we extend the length of the code to 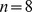, the improvement of the code properties are apparent. BC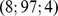 results in the largest set, together with perfectly matched experimental properties, but it can still correct only one substitution error. If we relax the experimental constraints slightly, two substitution errors are correctable, e.g., for BL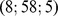 together with an acceptable set size.

Inspecting our upper bounds on the code size reveals that codes with even larger distance, might exist. According to the Singleton bound for 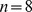 and 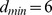, the upper bound on the code size is 64. But this already indicates, that we can never construct a code of size 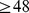 and length 8 that can guarantee to correct three errors, since in this case the upper bound on the code size is 16. This actually coincides well with the Hamming bound, which, for 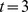 and 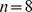, gives an upper bound on the code size of 14. Note that the random code RN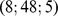 minimizes the error probabilities (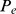 and 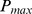).

Additional results on the distance distributions and error probabilities for equal set sizes, are presented in the *Supporting Information S1*. Selected barcodes (list of code words) can be found in the *ZIP file S1*.

## Discussion and Conclusion

It is shown, that compared to published barcodes, codes with similar length, larger cardinality and better error-correction capabilities (regarding substitution errors) exist, while retaining the experimental parameters of the Illumina barcode (which has length six and cardinality 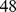). The latter may already be a reasonable choice for many applications, as, for example, the mean error introduced by the channel given by [15] is roughly 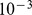, hence, one out of 1000 reads is wrongly demultiplexed. However, the maximum error of the Illumina barcode over this channel is very poor compared to even short codes of length 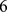. For applications being sensitive for such errors, much better short codes exist. For example, using a code of length 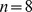, wrong demultiplexing occurs with one magnitude less. This rate of 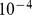 now approaches the rate at which indels occur. Consequently, in order to further increase the reliability of demultiplexing, codes have to be designed that are also able to correct indels. This means that considering the Hamming distance only and increase of the code length is not sufficient. Hence, the construction methods discussed in this paper can not be applied any more, as our randomized construction method has an exponentially increasing computational complexity.

As mentioned, we focused on the construction of codes with large cardinality given experimental constraints. It is quite interesting to note that for code lengths 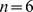 and 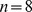, no codes with the comparable cardinality and better minimum distance can exist even if the experimental constraints are relaxed. For 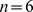 we present codes with minimum distances 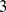 and 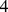. The latter is based on the currently best linear code, while the former was found by the randomly construction providing perfect experimental parameters. For slightly worse experimental parameters, our randomly constructed code provides the largest barcode set. Regarding the minimum distance the construction based on linear codes is optimal as the cardinality of a code with minimum distance 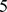 can not be larger than 16 (by the Singleton bound). For 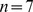 there is possibly room for an improvement as both Hamming and Singleton bound allow codes with a minimum distance of five, while the best codes we found have only minimum distance four. For all investigated experimental constraints barcrawl results in the largest barcode sets. Perfect experimental parameters and large barcode sets can be achieved for codes of length 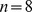 at the cost of a smaller minimum distance. At this point the user has to balance set size against error correcting capabilities.

Finally, let us note that both, design and decoding, assume a memoryless and symmetric channel. Since real applications deviate from this behaviour, we compared our barcodes using a non-symmetric channel.

In this paper we compared published barcodes with our own codes and presented advantages and disadvantages of the different sets. It is now up to the biological user to choose the best barcode set for each individual application.

## Supporting Information

Information S1
**Additional Data and Figures.** Comparison of barcode sets with different lengths and experimental constraints. Each set is reduced to an equal set size of 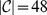.(PDF)

ZIP file S1 **File of barcode sets. This file contains a list of all barcodes considered in the paper.**
(ZIP)
