# Supplementary material for: Short Barcodes for Next Generation Sequencing
Source: PLoS One. 2013 Dec 30;8(12):e82933. doi: 10.1371/journal.pone.0082933 (PMC3875435; doi:10.1371/journal.pone.0082933)
Supplement: Information S1 — Additional Data and Figures. Comparison of barcode sets with different lengths and experimental constraints. Each set is reduced to an equal set size of . (PDF) [file pone.0082933.s001.pdf]

Short Barcodes for Next Generation Sequencing

Katharina Mir[1], Klaus Neuhaus[2], Martin Bossert [1] and Steffen Schober[1,†],
1 Institute of Communications Engineering, Ulm University, Albert-Einstein-Allee 43, 89081 Ulm, Germany
2 Lehrstuhl für Mikrobielle Ökologie, Wissenschaftszentrum Weihenstephan, Technische Universität München Weihenstephaner Berg 3, 85350 Freising, Germany
† Corresponding author: steffen.schober@uni-ulm.de

Compare barcodes sets of same size

In the following, we compared our randomly drawn barcodes, RN(n; $|\mathcal{C}|$; d_{min}) (each code is the best found in 100 trials) with published barcodes. We considered the codes proposed by Bystrykh [1, Supplementary Table S2 (Page 5,6)] of different lengths which are denoted with BY(n; $|\mathcal{C}|$; d_{min}) and the codes obtained by barcrawl with BC(n; $|\mathcal{C}|$; d_{min}). We further considered the BCH code of length 8 and $d_{min} = 4$ used by Krishnan *et al.* [2], denoted by BCH(n; $|\mathcal{C}|$; d_{min}).
Finally, we consider the best known linear codes, BL(n; d_{min}) over $GF(4)$ of different lengths n. Note that the dimension is $k \geq 3$ in order to get enough codewords. The best linear codes with the largest minimum distance can be downloaded via MAGMA [3]. Our goal is to compare the cardinalities of the barcode sets, therefore we fixed for different (n; d_{min}) the experimental constraints, the homopolymer length and the range of the GC content. The basic properties of all investigated barcodes are summarized in the paper (greedy approach for RN) or in Table S1 (non greedy approach).

For a fair comparison of the barcode sets with the Illumina set, we constrained the set size to $|\mathcal{C}| = 48$. Therefore, we chose the code words with the best experimental constraints until the desired set size is reached. The experimental parameters of a barcode can be improved by expurgating improper code words, especially, this is necessary for linear codes. This also allows to *trim* a code to a certain size. To this end, we construct a new barcode from a another one, by deleting all code words that do not meet the constraints.. In the table the codes that had to be shortened are denoted with $_{|\mathcal{C}|=48}$. The properties of the sets are presented in Table S2. Please note that during the expurgation, the set was not optimized to minimize the errors.

	$\|\mathcal{C}\|$	n	GC [%]	h_{max}	d_{min}	P_e	P_{max}	Information
IL(6; 2)	48	6	0-83.3	4	2	0.003720	0.024100	
BY(6; 3)	13	6	50	1	3	0.00115744974058	0.00152675606062	$\|\mathcal{C}\| < 48$
RN(6; 3)	50	6	50	1	3	0.00297930893355	0.00402310175229	
BC(6; 3)	45	6	50	1	3	0.00293934865265	0.00433319686588	$\|\mathcal{C}\| < 48$
BY(6; 3)	48	6	33.3-66.7	2	3	0.00287479186204	0.00344409742315	
RN(6; 3)	87	6	33.3-66.7	2	3	0.00343156224784	0.00568180648915	
BC(6; 3)	90	6	33.3-66.7	2	3	0.00333779055118	0.00479221839011	
BL(6; 4)	0	6	50	1	4	not calc.	not calc.	$\|\mathcal{C}\| < 48$
RN(6; 4)	18	6	50	1	4	0.00137070824428	0.00196252151513	$\|\mathcal{C}\| < 48$
BC(6; 4)	21	6	50	1	4	0.00170852113961	0.00223078114371	$\|\mathcal{C}\| < 48$
BL(6; 4)	60	6	33.3-66.7	2	4	0.00350044276484	0.00415971952076	
RN(6; 4)	24	6	33.3-66.7	2	4	0.00168122793162	0.00251614902219	$\|\mathcal{C}\| < 48$
BC(6; 4)	27	6	33.3-66.7	2	4	0.00213145291469	0.00308957132629	$\|\mathcal{C}\| < 48$
BY(7; 3)	52	7	42.9-57.1	1	3	0.00243615939854	0.00317400700813	
BL(7; 3)	34	7	42.9-57.1	1	3	0.001454780995	0.00234874403316	
RN(7; 3)	127	7	42.9-57.1	1	3	0.00396875564856	0.00577496868071	
BC(7; 3)	134	7	42.9-57.1	1	3	0.00409145276126	0.00585134792272	
BL(7; 4)	29	7	42.9-57.1	2	4	0.000889486214847	0.00192453915606	$\|\mathcal{C}\| < 48$
RN(7; 4)	59	7	42.9-57.1	2	4	0.00206484064768	0.00312652637977	
BC(7; 4)	67	7	42.9-57.1	2	4	0.00246956732624	0.00427006271525	
BL(7; 4)	41	7	28.6-71.4	2	4	0.00122876771354	0.00234288468511	$\|\mathcal{C}\| < 48$
RN(7; 4)	63	7	28.6-71.4	2	4	0.00217146534681	0.00343869381956	
BC(7; 4)	71	7	28.6-71.4	2	4	0.00270966900931	0.00398044085558	
BL(7; 4)	62	7	14.3-85.7	3	4	0.00188935248825	0.00299764003488	
RN(7; 4)	69	7	14.3-85.7	3	4	0.00240248986473	0.00392474941953	
BC(7; 4)	78	7	14.3-85.7	3	4	0.00291964205936	0.0048276024047	
BCH(8; 4)	4	8	50	1	4	0.000130877064386	0.000153526113743	$\|\mathcal{C}\| < 48$
BY(8; 4)	52	8	50	1	4	0.00138438837587	0.00174725711803	
BL(8; 4)	8	8	50	1	4	0.00017211463306	0.000435829557311	$\|\mathcal{C}\| < 48$
RN(8; 4)	88	8	50	1	4	0.00182308722335	0.00284337060676	
BC(8; 4)	97	8	50	1	4	0.00208718229848	0.00348552721763	
BL(8; 5)	50	8	37.5-62.5	3	5	0.000164930826698	0.000271101846767	
RN(8; 5)	48	8	37.5-62.5	3	5	0.000156396009809	0.000220646289528	
BC(8; 5)	50	8	37.5-62.5	3	5	0.000164977426615	0.000263832223954	
BL(8; 5)	58	8	25-75	3	5	0.000185250799755	0.000305507449322	
RN(8; 5)	48	8	25-75	3	5	0.000159346178181	0.000232780604067	
BC(8; 5)	56	8	25-75	3	5	0.000175939065403	0.00027766799098	

Table S1. Properties of barcode sets

Now a fair comparison regarding the error probabilities over an symmetric channel as well as an non-symmetric channel is possible. The distance profiles are helpful in order to understand some of the results.

	$\|\mathcal{C}\|$	n	GC [%]	h_{max}	d_{min}	P_e	P_{max}	Comparison
IL(6; 2)	48	6	0-83.3	4	2	0.003720	0.024100	Yes
$RN(6;3)_{\|\mathcal{C}\|=48}$	48	6	50	1	3	0.00292755079606	0.00402014853915	Yes
BY(6; 3)	48	6	33.3-66.7	2	3	0.00287479186204	0.00344409742315	Yes
$RN(6;3)_{\|\mathcal{C}\|=48}$	48	6	33.3-66.7	2	3	0.00298068022793	0.00438270013817	
$BC(6;3)_{\|\mathcal{C}\|=48}$	48	6	33.3-66.7	2	3	0.00290411957939	0.00389833000201	Yes
$BL(6;4)_{\|\mathcal{C}\|=48}$	48	6	33.3-66.7	2	4	0.00331005014859	0.0040199205281	Yes
$BY(7;3)_{\|\mathcal{C}\|=48}$	48	7	42.9-57.1	1	3	0.00224627608815	0.00317393832312	Yes
$RN(7;3)_{\|\mathcal{C}\|=48}$	48	7	42.9-57.1	1	3	0.00223057348631	0.00349388642128	Yes
$BC(7;3)_{\|\mathcal{C}\|=48}$	48	7	42.9-57.1	1	3	0.00226070316665	0.00363756423053	Yes
$RN(7;4)_{\|\mathcal{C}\|=48}$	48	7	42.9-57.1	2	4	0.00174420189638	0.0026693905228	Yes
$BC(7;4)_{\|\mathcal{C}\|=48}$	48	7	42.9-57.1	2	4	0.00191186099293	0.00354318382723	Yes
$RN(7;4)_{\|\mathcal{C}\|=48}$	48	7	28.6-71.4	2	4	0.00180337791026	0.00307063237823	
$BC(7;4)_{\|\mathcal{C}\|=48}$	48	7	28.6-71.4	2	4	0.00199406378921	0.0030065889882	
$BL(7;4)_{\|\mathcal{C}\|=48}$	48	7	14.3-85.7	3	4	0.00147031593282	0.00229922173227	Yes
$RN(7;4)_{\|\mathcal{C}\|=48}$	48	7	14.3-85.7	3	4	0.00178307754203	0.0028839028965	
$BC(7;4)_{\|\mathcal{C}\|=48}$	48	7	14.3-85.7	3	4	0.00203845992754	0.00308250540922	
$BY(8;4)_{\|\mathcal{C}\|=48}$	48	8	50	1	4	0.00129829898932	0.00174692777369	Yes
$RN(8;4)_{\|\mathcal{C}\|=48}$	48	8	50	1	4	0.00109440649891	0.00198494873607	Yes
$BC(8;4)_{\|\mathcal{C}\|=48}$	48	8	50	1	4	0.00111233207692	0.00213645973667	Yes
$BL(8;5)_{\|\mathcal{C}\|=48}$	48	8	37.5-62.5	3	5	0.000160616844003	0.000266116197645	Yes
RN(8; 5)	48	8	37.5-62.5	3	5	0.000156396009809	0.000220646289528	Yes
$BC(8;5)_{\|\mathcal{C}\|=48}$	48	8	37.5-62.5	3	5	0.000162385883016	0.000261783546578	Yes
$BL(8;5)_{\|\mathcal{C}\|=48}$	48	8	25-75	3	5	0.000162435680132	0.000280923879543	
RN(8; 5)	48	8	25-75	3	5	0.000159346178181	0.000232780604067	
$BC(8;5)_{\|\mathcal{C}\|=48}$	48	8	25-75	3	5	0.00016315305713	0.000255397281183	

Table S2. Properties of barcode sets (shortened to) same cardinality. The average (P_e) and the maximum error (P_{max}) are obtained over a non-symmetric channel defined in the *Channel Model* part.

For comparison we determine for each construction method and for each (n, d_{min}) the set which has the smallest mean error over the non-symmetric channel.

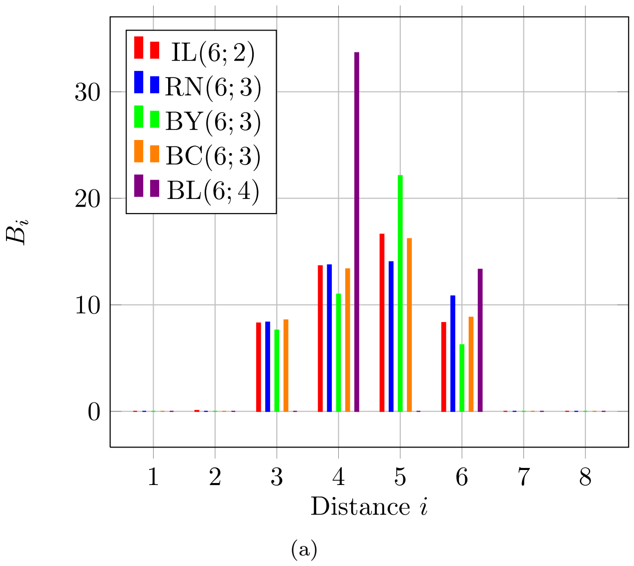

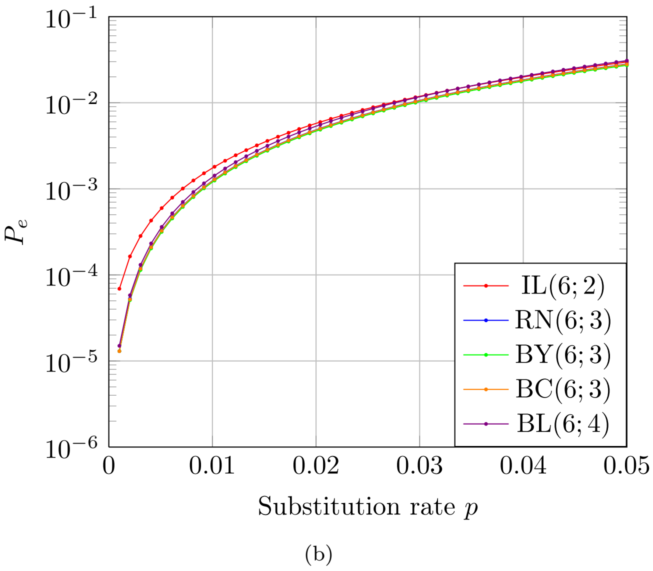

Figure S1. Distance distribution and average decoding error probability for barcodes of length 6 over the symmetric channel.

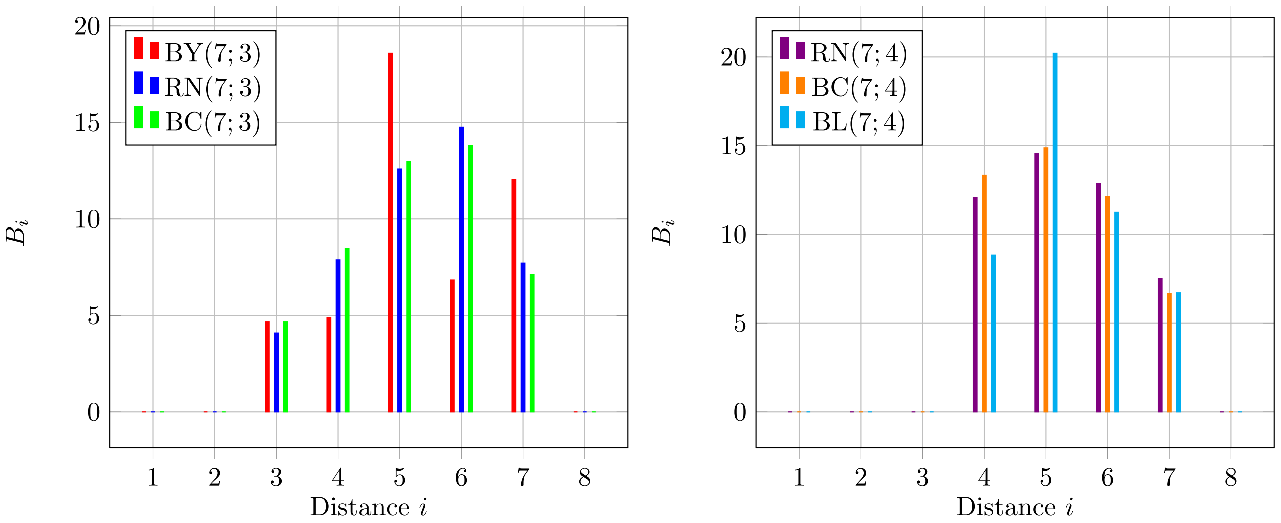

(a) (b)

Figure S2. Distance distribution for barcodes of length 7.

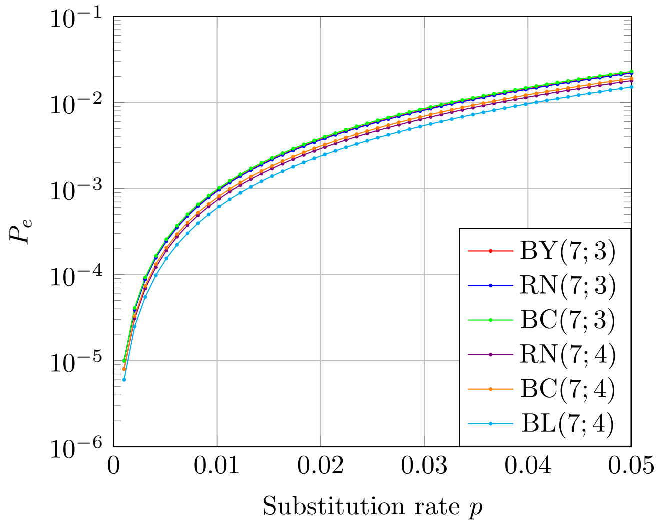

Figure S3. Average decoding error probability for barcodes of length 7 over the symmetric channel.

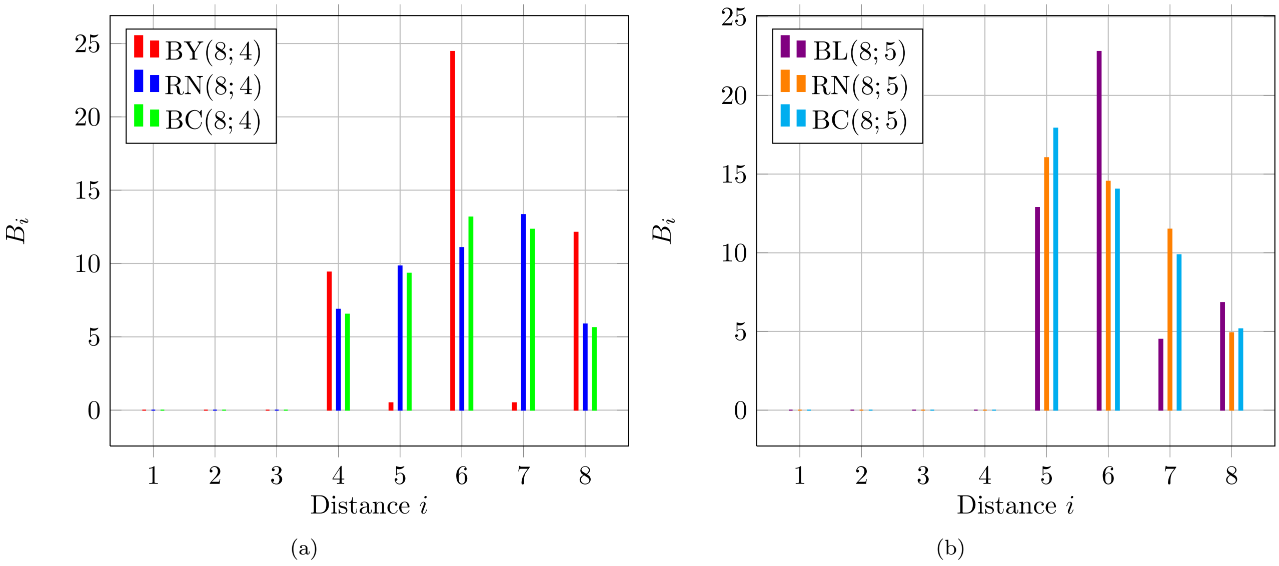

Figure S4. Distance distribution for barcodes of length 8.

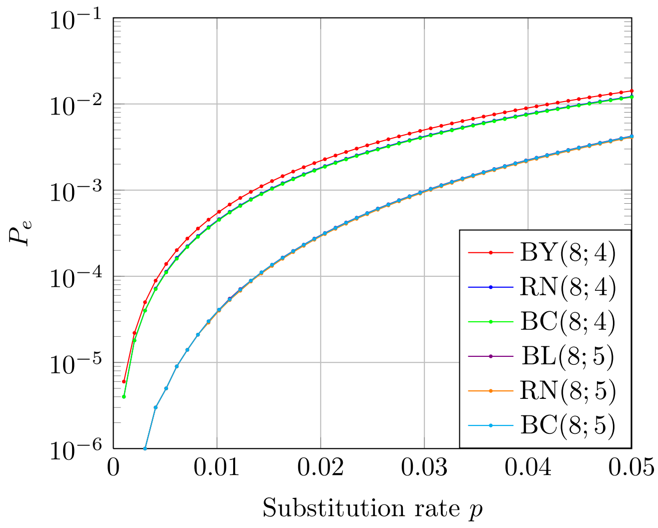

Figure S5. Average decoding error probability for barcodes of length 8 over the symmetric channel.

References

1. Bystrykh LV (2012) Generalized DNA barcode design based on hamming codes. PLoS ONE 7: e36852.

2. Krishnan A, Sweeney M, Vasic J, Galbraith D, Vasic B (2011) Barcodes for DNA sequencing with guaranteed error correction capability. Electronics Letters 47: 236 –237.

3. Bosma W, Cannon J, Playoust C (1997) The Magma Algebra System I: The User Language. Journal of Symbolic Computation 24: 235-265.
